# Improving algebraic understanding using history of mathematics: the case of structural reasoning in cubic equations

**DOI:** 10.3389/fpsyg.2026.1788990

**Published:** 2026-04-30

**Authors:** Alfred Gyasi Bannor, Joseph Frank Gordon, Yarhands Dissou Arthur

**Affiliations:** Department of Mathematics Education, Akenten Appiah-Menka University of Skills Training and Entrepreneurial Development, Kumasi, Ghana

**Keywords:** algebraic structure, cubic equations, history of mathematics, mathematics education, reification, structural reasoning

## Abstract

Teaching algebra at the senior high school level often privileges procedural fluency at the expense of deeper conceptual understanding, which has left students unable to reason about the underlying structures of algebraic objects. This study investigates the potential of integrating the history of mathematics (HoM) as a pedagogy to foster students’ structural reasoning in cubic equations. Grounded in the structural reasoning framework of Harel and Soto, the study employed a quasi-experimental non-equivalent control group design involving 128 senior high school students from two schools in Ghana. The experimental group received history-integrated lessons on cubic equations, drawing on historical developments such as the classification of cubic forms, transformations to depressed cubics, and early solution methods, while the control group received standard curriculum-based lessons. Quantitative data were collected using structural reasoning test and analysed using Quade’s non-parametric ANCOVA to control for pre-existing differences. Results revealed statistically significant and practically meaningful difference in structural reasoning between groups. Qualitative data collected using semi-structured interviews further illuminated how historical contexts enhanced students’ recognition of algebraic structure, deepened conceptual understanding, and shifted problem-solving approaches from procedures execution to structural analysis. The findings provide empirical evidence that the HoM can function as more than an enrichment tool; it can serve as a powerful instructional resource for promoting advanced algebraic thinking. This study contributes to research in history-and-pedagogy-of-mathematics by demonstrating how historically informed lessons can support structural reasoning in higher-order polynomial contexts and offers a theoretically grounded model for integrating HoM into secondary algebra teaching.

## Introduction

It is rather unfortunate that teaching algebra at the senior high school has emphasised mostly on developing procedural proficiency at the expense of deeper conceptual understanding (Apawu et al., 2018; Susac et al., 2014). On one hand, it makes more sense when it is argued that the mathematics curriculum is assessment driven; so, students need procedural and algorithmic skills to perform higher in assessment tasks. Thus, teachers need to teach students the skills they might need to tackle problems step by step. Students should be able to manipulate, operate on or simplify algebraic expressions and solve equations. On the other hand, it is worthwhile noting that, despite high procedural abilities found in students, they may not have profound in-depth grasp of algebraic concepts. This can result to students developing poor understanding about the inherent structures that govern algebraic representations (Harel, 2013). According to Harel and Soto (2017) teaching students to be fluent in performing procedural tasks alone might not translate into strong algebraic reasoning ability. Hence, the need for pedagogical considerations.

Ordinarily, procedural proficiency often masquerades as profound algebraic understanding (Kieran, 2013). However, genuine algebraic understanding is informed by the ability to reason about and make use of algebraic structures (Kaput, 2008; Blanton and Kaput, 2011; Sibgatullin et al., 2022). Structural reasoning which is also called “structure sense” (Linchevski and Livneh, 1999; Novotná and Hoch, 2008) defines algebraic thinking beyond what the student can just be able to do to solve equations or operate on algebraic expressions. On this note, the concept of structural reasoning is defined in the present study to involve students’ ability to recognise algebraic expressions, functions and equations as composed of meaningful substructures or entities (Harel, 2013). Instead of seeing algebraic forms to simply comprise of sequences of symbols and letters to be operated upon, structural reasoning is the students’ ability to recognise structures in algebraic forms. This ability is what informs students skills to factor, identify patterns, see equivalences or identify invariants (Sfard, 1991). The cultivation of structural reasoning is, thus, seen as central to algebraic proficiency (Harel and Soto, 2017). Hence, calls have been made to look for effective ways to develop it within students (e.g., Harel, 2018). It is in the ambits of this that the present study looks at the utility of integrating the history of mathematics (HoM).

The position of the HoM in mathematics education has received increasing attention in the literatures quite recently (Chorlay et al., 2022). The HoM is the study of and use of historical antecedents behind mathematics concepts to improve on mathematics teaching-learning (Karp and Furinghetti, 2016). According to Jankvist (2009), the HoM can be used as a tool where it serves to enrich mathematics instruction. By this, it provides students with meaningful “genetic” contexts that illuminate how mathematics concepts developed to become what we know of today (Farmaki et al., 2004). It is found that integrating the HoM sources into classroom helps students see mathematics not as a field of fixed body of cumulative knowledge but as an evolving discipline shaped by human discovery and creativity (Doğruer, 2024; Liu, 2003; Radford et al., 2002). The pedagogy of HoM fosters reflection on the origins, evolutions, and purposes of mathematics concepts. This makes abstract concepts seem more concrete, accessible and meaningful to students (Arthur et al., 2025). In this theory, the HoM does more than recount past stories or discoveries; it becomes a pedagogical bridge that links the cognitive development of students to the epistemological dimension of mathematics concepts (Fried, 2014).

HoM offers powerful contexts to mathematics teaching and learning in which students can appreciate why algebraic concepts and methods invented to deal with them emerged and how algebraic forms developed and settled into coherent structures (Katz and Barton, 2007; Radford, 2001). The authors of this paper hold the premise that by engaging students with historical contexts when teaching higher order polynomial equations, they may see learning not just as mastering procedures or algorithms to follow to arrive at a solution, but recognise such equations as objects shaped around meaning, form, generalisation and structure. This premise is supported by the genetic method (Farmaki and Paschos, 2007; Tzanakis and Arcavi, 2002) where proponents suggest that integrating original “embryonic” sources in the HoM can provide epistemological justification of mathematics objects and the intellectual motivation behind them (Barbin, 2013; Clark, 2019; Radford, 1997; Swetz, 1995). As a matter of fact, this study tends to focus on how using the history behind cubic equations could improve students reasoning about the structural relationships that exist within cubic expressions and equations.

Been motivated by the thesis of Lichy (2025) that HoM can be used to foster pre-algebraic thinking, we proposed that HoM may present fertile grounds for students structural reasoning, which is advanced form of algebraic thinking. In this paper, our focus is specifically on using cubic equations as the instructional context for the integration of HoM. We suggest that by teaching students how to identify depressed cubic forms, perform transformations, factor forms, and apply ancient methods to solve equations, as all were done in the HoM, they may develop good recognition of the cubic structure. By doing so, the present study fills gaps that intersects research in the history and pedagogy of mathematics (HPM), algebraic cognition, and pedagogical practice in mathematics education. First, it involved investigating how teaching a lesson designed around the history of cubic equations could improve students’ structural reasoning in cubic equation, an area that is not studied before in the literatures. Second, this study answers the question of how HoM can be used as a context for teaching higher-order polynomials. This addresses a significant gap in the literature by overturning the focus of HoM integration studies in algebra teaching that lean towards simple concepts such a square root (Goktepe and Ozdemir, 2021), logarithm (Panagiotou, 2011), etc. Finally, the study offers a model for how HoM interventions can support the development of deeper algebraic thinking and contributes to an understudied instruction context of cubic equations in mathematics education research.

### Problem statement

Despite the emphasis on teaching senior high school students deeper algebraic understanding (e.g., Apawu et al., 2018), its development remains uneven and protracted (Sun et al., 2023; Susac et al., 2014). Most at times, students become trapped between procedural fluency and full structural objectification of concepts (e.g., Castro et al., 2025). The result is that students can perform symbolic operations fluently yet fail to notice the deeper algebraic relationships that give meaning to those operations (Sfard, 1991; Torres et al., 2021; Torres et al., 2025). This issue is particularly seen by the first author in the study of cubic equations. Per his own experiences as at the senior high school, he had witnessed a number of instances where students may competently factorise and find roots using the rational root test or by drawing graphs but fail to recognise the relationships among coefficients, roots, and overall polynomial structure that give insights to the solutions of the cubics. When solving students were given $x^{3}-6x^{2}+11x-6=0$ to solve, for example, many obtained the correct factorisation using the rational root test and long division as $(x-1)(x^{2}-5x+6)=0$. Yet, when they were asked about what are the relationships that exist among the features of the structure of the equations, they could not identify that the sum of the roots equals the coefficient of *x*^2^ with opposite sign or that the product of the roots equals the constant term with opposite sign. They treated cubic equations as just to be worked with a sequence of procedures without any insights about what structures lie beneath them. This might stem from students not having an intuitive taught of the structural properties inherent in algebraic concepts.

While this difficulty manifests most visibly at the student level, it is also deeply rooted in systemic instructional practices of teachers. Especially when teachers’ design instruction such that classroom lessons privilege procedural competence over algebraic understanding. Teachers constrained by standard curriculum emphasise students grasp of procedures rather than teaching students to reason about underlying patterns and structural relationships with concepts. Given this persistent problem, there is a pressing need for pedagogical interventions that integrate meaning to structure in algebra learning. Recent research has explored the potential of pattern recognition, generalisation, and multi-representational learning (e.g., Blanton et al., 2019; Godino et al., 2017); however, one promising and underutilised path lies in the integration of the HoM as a means to illuminate the understanding of algebraic structure. Scholars contend that when teachers retrace the history of concepts and how it has transformed historically enables students to experience, at a cognitive level, the same processes of abstraction and structural generalisation that shaped algebraic concepts (Fried, 2018; Katz and Barton, 2007; Radford, 2014; Sfard, 1995). This study therefore addresses the problem of difficulty in enhancing structural reasoning by using the historical context of cubic equations to make algebraic structures visible, meaningful, and intellectually coherent.

The synthesis of history and cognition responds to Harel and Soto (2017) call for instructional designs that promote reasoning about structure and to the advocacy for historically informed mathematics lessons. Despite the richness of the literatures in algebraic cognition, and the steadily growing body of papers on history-in-mathematics education, several gaps remain. First, few studies explicitly target cubic equations as the instructional context for linking history (Baki and Guven, 2009). Second, while many interventions address conceptual and procedural understanding, less attention has been paid to structural reasoning as a learning outcome in cubic equations, particularly in senior high school algebra settings. Third, although historical interventions are advocated, empirical evidence about how history supports structural reasoning is sparse.

Therefore, this study aims to fill these gaps by designing and investigating an intervention around the history of cubic equations, with measures of structural reasoning as outcomes. To achieve this goal, the study sought to answer the following research questions: (1) To what extent does participation in history-integrated lessons on cubic equations improve senior high school students’ structural reasoning performance compared to students receiving traditional instruction? (2) How do students in the history-integrated lessons describe their learning experiences and reasoning processes?

## Literature review

### Theoretical framework

#### Structural reasoning

What the term ‘structure” is actually all about could mean differently across different contexts and individual perspectives (Hoch and Dreyfus, 2006; Stehlíková, 2004; Harel and Soto, 2017). In a general sense, a structure may be understood as an entity composed of multiple parts arranged or put together in a particular way to form a coherent whole (Psillos, 2006). In relation to mathematics, when talked about in the context of algebra, this image of structure becomes especially relevant in senior high school mathematics. Here, algebraic expressions, equations, and functions are often discussed in terms of their internal organisation (Hoch, 2003). Pertaining to this view, structure arises through the relationships the student conceives among different algebraic components (Sfard and Linchevski, 1994). Importantly, the way these components are connected to one another is not limited to physical or spatial configurations, but can involve abstract or conceptual arrangements as well (Harel and Soto, 2017). An algebraic expression, $ax^{2}+\mathrm{bx}+c=0$, may be regarded as having a particular structure defined by its roots, coefficients, intercepts, turning points, line of symmetry, etc., when it is interpreted as an ordered string of symbols intentionally arranged to convey specific mathematical meaning. This way, algebraic structure is portrayed in terms of both the shape and the order that binds the constituent parts of algebraic representations.

Students’ knowledge of and ability to think about and make good use of this structure is what is termed as structural reasoning (Harel and Soto, 2017). The idea that students should *“look for and make use of structure”* has been emphasised in the Common Core State Standards in Mathematics (CCSM) since 2010 in the United States of America. While the recent standards-based curriculum in Ghana is an adaptation of some if not all the standards in the CCSSM, it implies that reasoning about structure is also important in learning the algebra strand. Though, attaining full structural reasoning among students is not clearly emphasised as a competence in the new additional mathematics curriculum, rather it is emphasised on students to have the ability to breakdown concepts into constituent parts and make meaning from them. It is stated that students should develop *“The ability to break things down into their parts and determine relationships between those parts and being able to tell the difference between what is relevant and irrelevant ………. Breaking material into its constituent parts and detecting how the parts relate to one another and an overall structure …. is required. …….”* (Ministry of Education (MoE), National Council for Curriculum and Assessment, 2023). This statement is analogous to what the CCSSM (Association Center for Best Practices and Council of Chief State School Officers, 2010) point to. In the literature, it has been a major expectation that the formation of students’ structural reasoning must begin long before they enter college (e.g., Harel and Soto, 2017). So, throwing more light on it at the senior high school level is, thus, very promising.

Theoretical assertions all support the claim that the development of structural reasoning is a priority. Sfard (1991) posits that learning algebra should transition from viewing symbolic operations as processes to conceiving concepts as objects with structures upon which further reasoning can occur. Within this lens, reasoning about structure represents the consolidation of algebraic concepts as manipulable objects (Marghetis et al., 2016). Hawthorne and Druken (2019) in their perspective stresses that algebraic understanding should also comprise the ability to discern, interpret, and utilise the underlying form of algebraic expressions and relations (Kieran, 2018). Then, it is not just recognition of the form but the ability to coordinate representational systems to reveal invariant properties (Duval, 2006). Authors (e.g., Kieran, 1992; Kaput, 1998) believe that algebraic power lies in the student’s ability to identify underlying structures and relationships with algebraic representations. Hence, true algebraic understanding does not only rest on the ability to execute procedures but more on the ability to identify and utilise the structures inherent in algebraic representations.

**Figure 1:**
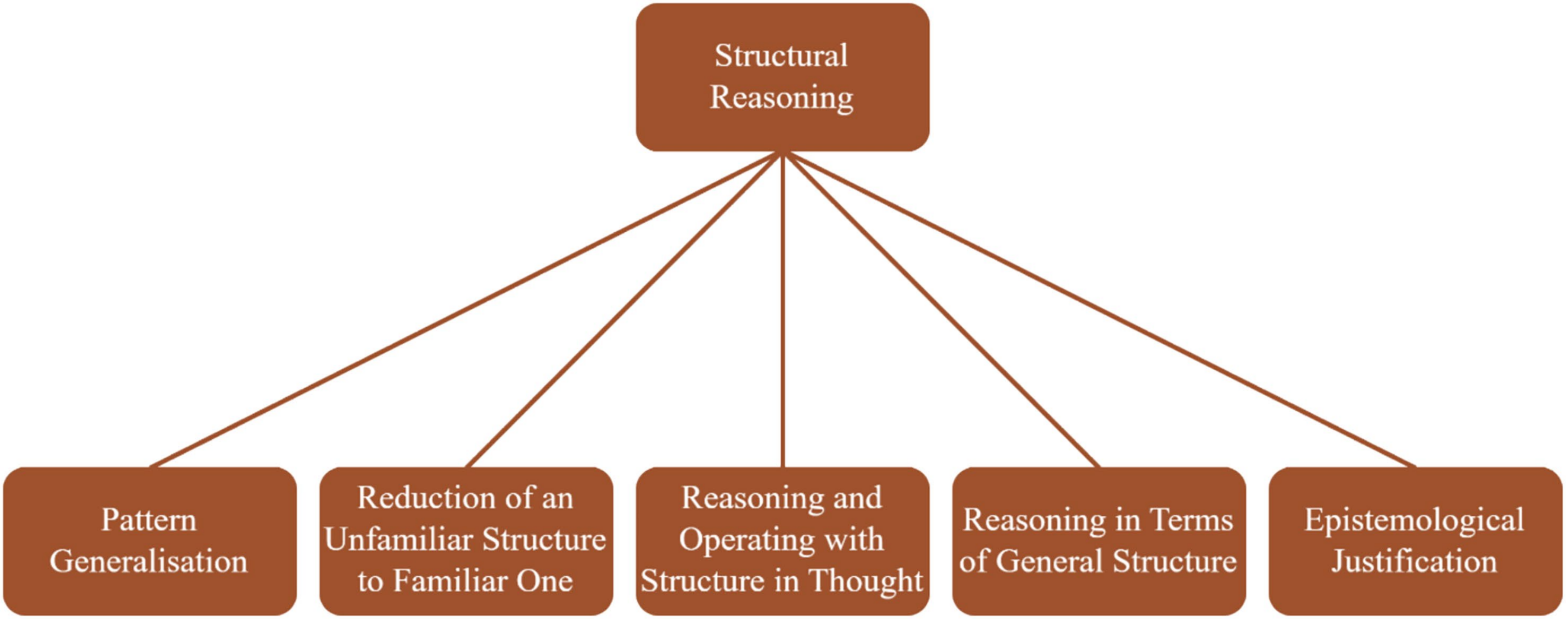
Dimensions of structural reasoning (Harel and Soto, 2017).

Although the term structural reasoning is intuitively meaningful, it proves difficult to define precisely because it appears in many different forms across mathematical activity (Harel and Soto, 2017). It might be through its theoretical perspective that we can see how the true nature of structural reasoning can be. To some authors (e.g., Küchemann and Hoyles, 2009), structural reasoning is closely aligned with formal deductive reasoning. According to Küchemann and Hoyles (2009), it is the students’ capacity to infer and deduce from mathematical structures. A more coherent view of structural reasoning points to it to attend to form: to the patterns, sub-structures, equivalences, transformations embedded in algebraic forms (e.g., Harel, 2013; Hoch and Dreyfus, 2006; Linchevski and Livneh, 1999). Earlier Harel (2013) defined the concept as merely an act of relating and with this opening or limited definition he sets structural reasoning as the combined ability to: (a) look for structures, (b) recognise structures, (c) probe into structures, (d) act upon structures, and (e) reason in terms of general structures.” Later on, Harel and Soto (2017) expanded the definition to include one further aspect of structural reasoning, which is (f): the ability to see (be aware of) how a piece of knowledge acquired resolves a “perturbation experienced,” what is dubbed in Harel (2013) as epistemological justification (See Figure 1 for the typology). The theoretical framework of structural reasoning offered by Harel and Soto (2017) is, thus, what underpins this study. In the next paragraphs, we discuss the key headings (as in Harel and Soto, 2017) of each of these dimensions and use examples to explain how they manifest when dealing with the concept of cubic equations.

##### Pattern generalisation

Harel and Soto (2017) identify pattern generalisation as one of the major dimensions of structural reasoning. They emphasised it is the student’s ability to transition from identifying regularities to articulating general structure of relationships. They distinguish between two dimensions which are: (i) result-pattern generalisation, in which students generalise based solely on repeated outcomes, and (ii) process-pattern generalisation, in which students generalise by discerning regularities in the operations, transformations, or structural mechanisms that produce certain outcomes. According to Harel and Soto, the transition from merely observing recurring outcomes to understanding and articulating the structures that generate them is essential for developing genuine mathematical structure sense. This theoretical distinction can become especially visible in students’ understanding with cubic equations. Students first encounter cubic equations through graphical investigations. A student who notices, across several examples such as $x^{3}-6x+4=0$, $2x^{3}+x-1=0$, and $x^{3}+x^{2}-x-1=0$, that the equations frequently exhibit turning points or tend to possess at least one real root is engaging in result-pattern generalisation. Students could infer a pattern simply from observed outcomes. This type of generalisation, while a meaningful early step, does not yet involve reasoning about why such regularities occur. By contrast, process-pattern generalisation emerges when students begin to analyse the structural features that govern the behaviour of cubic forms. For instance, students who investigate how the shape of the cubic graph, and consequently the number of real roots depends on properties such as the sign of the leading coefficient, the presence of an inflection point, or the nature of the discriminant, start to generalise from the processes underlying the examples rather than from outcomes alone. A student who recognises that the presence of both a local maximum and a local minimum is what allows some cubics to intersect the *x*-axis three times is engaging in process-pattern generalisation. The generalisation here is no longer that “many cubics seem to have three real roots,” but rather that the existence of two critical points and their relative values determine whether a cubic will have one or three real roots. This generalisation is based on process, not outcome.

##### Reduction of unfamiliar structure to familiar one

Hoch and Dreyfus (2006) focused on this dimension of structural reasoning in their paper where they described structure sense in the context of high-school algebra as the ability to: (i) perceive an algebraic statement as a coherent entity, (ii) recognise it as a form previously encountered, (iii) decompose it into meaningful substructures, (iv) identify relationships between different structures, (v) determine which manipulations are possible, and (vi) decide which manipulations are strategically useful. Linchevski and Livneh (1999) defined structure sense as the ability “to use equivalent structures of an expression flexibly and creatively.” Kieran (1989) distinguished surface structure which is the visible form of an expression from systemic structure which is the underlying relationships of operations and their properties. Structural sense enables students to treat expressions as objects to be analysed, decomposed, and transformed rather than simply executing step-by-step algorithms. This includes recognising factorable patterns, seeing equivalence across apparently dissimilar expressions, and reasoning about general properties of algebraic objects rather than isolated instances. For example, when working with cubic equations in the general form of $x^{3}+bx^{2}+\mathrm{cx}+d=0$, the transformation x=y−b/3is needed to reduce it into the form of the depressed cubic $x^{3}+\mathrm{py}=q$ before solving. Mathematicians found it easier to work with this form but not the former. This shows how important it is to reason enough to be able to reduce unfamiliar algebraic structures into forms that you are familiar with before pursuing with any known operation.

For example, it was very difficult to solve $x^{3}+6x^{2}+11x+6=0$ but it becomes more solvable when its depressed. Let us walk through this example to see how ancient mathematicians went by this transformation.

First, set:


$$x=y-\frac{b}{3a}=y-\frac{6}{3(1)}=y-2$$

Substituting into the equation, we have:


$${\left(y-2\right)}^{3}+6{\left(y-2\right)}^{2}+11(y-2)+6=0$$

Expanding:

$$y^{3}-6y^{2}+12y-8+6y^{2}-24y+24+11y-22+6=0$$

Grouping like terms and simplifying:

$$y^{3}-6y^{2}+6x^{2}+12y-24y+11y-8+24-22+6=0$$

$$y^{3}-y=0$$

You can see that the final equation looks more simplified than the original equation but roots may remain unchanged when solved. This is how transformations work and what remains unchanged is called the invariant.

##### Recognising and operating with structure in thought

This form of reasoning resonates with what Dubinsky (1991) referred to as “process conception.” It involves the ability to mentally operate on algebraic expressions without actually working them with the hand. This ability to manipulate and reason about structures mentally can be illustrated through looking at the general cubic equation. Students can check if the sum of the coefficients of $x^{3}+bx^{2}+\mathrm{cx}+d=0$ which is 1+b+c+dis 0. If that is the case then without tangible solving or doing any factorisation, students can say that x=1 is a root. For example, given $x^{3}-4x^{2}+x+2=0$, the sum of the coefficients: 1+(−4)+1+2=0. This implies directly that x=1 is a root of the equation. Also, the sign of *a* tells whether the solving the equation should expect one real root or three real roots. Looking at these examples, it suggests that students should be able to both form a nested structure and make a logical conclusion in thought. They can express their reasoning without accompanying it with written work showing symbolic representation (Dubinsky and McDonald, 2001).

##### Reasoning in terms of general structure

Instead of reasoning only in terms of the instances of algebraic structures, Harel and Soto (2017) theorise that this dimension manifests in the sense that students should demonstrate the ability to reason in terms of general structures. Harel (2013) has split this way of reasoning in terms of conceptual entities and reasoning in terms of operations on conceptual entities. Harel and Soto (2017) cited the APOS theory (Dubinsky and McDonald, 2001) and the idea of concept image (Tall and Vinner, 1981) as the foundation of this reasoning. If the student has formed a concept image of an algebraic entity, then reasoning about its general structure will not be a problem (Tall and Vinner, 1981). For this to happen, first, the student may form an image of the concept in a way that seems concrete and then base on this to abstract it into an instance of mathematical structure. To Dubinsky and McDonald (2001), this can only occur if the student conceives algebraic objects as conceptual entities. For students to be able to reason in terms of general structures, there are some epistemic processes that underlie this. Dorier and Sierpinska (2001) speaks about two processes that are key. With examples in reasoning about general structures in cubic equations, we highlight these processes: (i) Recognising similarities and generalising concepts, where students work with several specific cubic equations, such as $x^{3}-3x+2=0$, $x^{3}-\frac{1}{2}x^{2}-\frac{1}{2}x+\frac{1}{2}=0$, and $x^{3}+x^{2}-4x-4=0$, they may begin to notice shared properties: all are degree-three polynomials, their graphs have one inflection point, and the number of real roots depends on certain combinations of coefficients. By observing these similarities, students begin to unify these examples under the broader concept of cubic equations and generalise structural properties such as the role of the discriminant in predicting the number of real roots; (ii) making the concept explicit and reorganising knowledge, where once students consider the general cubic equation $x^{3}+bx^{2}+\mathrm{cx}+d=0$, they can reason about how the coefficients 1, *b*, *c*, *d* influence important structural features, including the discriminant, the number and type of roots, the relationships among roots (sum = −*b*; product = −*d*), and the end behaviour of the graph. This enables students to predict properties of any cubic equation and to reason structurally about its behaviour without needing to solve every individual case.

##### Epistemological justification

According to Harel and Soto (2017), epistemological justification refers to “a meta-cognitive awareness in which a student recognises how a newly acquired idea resolves an intellectual difficulty that they previously struggled with.” (Harel, 2018). This involves understanding the causal link between one’s confusion and the conceptual clue that clears it up, a process especially relevant when doing or interpreting mathematical proofs (Leron, 1985). Leron (1985) theorises that epistemological justification exposes the thinking behind proofs. It reorganises the traditionally linear presentation of proofs into a structure that reveals the hidden ideas and decision points that guided their creation. In dealing with cubic equations, this kind of reasoning appears when solving the general cubic. It is very easy to solve equations of the form of $x^{3}+\mathrm{mx}=n$, but solving $x^{3}+bx^{2}+\mathrm{cx}+d=0$ is very difficult in its form. The clue that lies here is using the substitution of x=y−b/3. Understanding why this approach worked rather than merely copying or following the steps reflects epistemological justification (Leron, 1985), because it reveals how a conceptual leap resolved a longstanding mathematical obstacle.

### History of mathematics

Advocacy for the integration of the HoM into mathematics classrooms has grown after several decades of research (Chorlay et al., 2022). The International Congress on Mathematical Education (ICME) and the European Summer University on the History and Epistemology in Mathematics Education (ESU) have shed considerable light on the history and pedagogy of mathematics (HPM) perspective (e.g., Clark et al., 2016; Barbin, 2013; Barbin and Tzanakis, 2014; Clark et al., 2019). HPM places emphasis on the interplay of history, mathematics, and education as three distinct yet productively interrelated dimensions that can enrich both the teaching and learning of mathematics and learning about mathematics (Clark et al., 2019). The HPM perspective argues that “perceiving mathematics both as a logically structured collection of intellectual products and as processes of knowledge production should be the core of the teaching of mathematics” (Clark et al., 2016). With this, the HPM perspective supports the idea that the HoM can be an engaging, thought-provoking, and valuable pedagogical resource of meaningful mathematics lessons.

According to Jankvist (2009), the HoM can be used both as a tool and as a goal. The first perspective which treats the HoM as a goal emphasises its value as a subject worthy of study in its own right (Jankvist, 2009). Meanwhile, this paper is founded on the alternative view of the HoM as a tool. This perspective focuses on integrating the HoM elements in mathematics lessons typically to enhance teaching and learning outcomes (Jankvist, 2009). Scholars argue that history can provide meaningful contexts, humanise mathematics, support teacher education, and deepen students’ understanding of why mathematics concepts were developed (Arthur et al., 2022; Bannor et al., 2024; Bütüner et al., 2020; Doğruer, 2024; Goktepe and Ozdemir, 2021; Kapofu and Kapofu, 2020; Lim and Chapman, 2015). The literature suggests that using the history of concepts can support mathematical understanding either directly or indirectly. Conceptually, by situating ideas in their original forms and explaining why strategies exist; structurally, by exposing students to how algebraic forms evolved, how generalisations came about, and how mathematicians thought about structure.

The integration of original historical sources into mathematics teaching is grounded in the theory that the epistemological evolution of mathematics ideas parallels the cognitive development of students (Sfard, 1991; Fried, 2018). This teaching philosophy views history not merely as resources for enriching teaching and learning context but as a means to reenact conceptual breakthroughs that illuminate the nature of mathematics reasoning. Katz and Barton (2007) contend that engaging with historical sources fosters a meta-mathematical awareness which refers to understanding of how mathematics knowledge is constructed, represented, and justified. In view of cultural-semiotic theory, Radford (2014) reinforces this position where he suggests that algebraic reasoning emerges through the progressive development of semiotic systems. The historical trajectory from rhetoric and geometric reasoning to symbolic algebra represents not only a change in notation and representation but also a transformation in the structure of reasoning itself. When students reconstruct historical mathematics arguments, they experience analogous transitions, moving from concrete reasoning about quantities to abstract reasoning about structures. Fried (2018) reports that students exposed to historical problem contexts exhibit greater engagement and more reflective reasoning. Other studies demonstrate that historical case studies can serve as cognitive bridges, helping students internalise abstract ideas by situating them within meaningful epistemic narratives (Radford, 2014; Katz and Barton, 2007).

**Figure 2:**
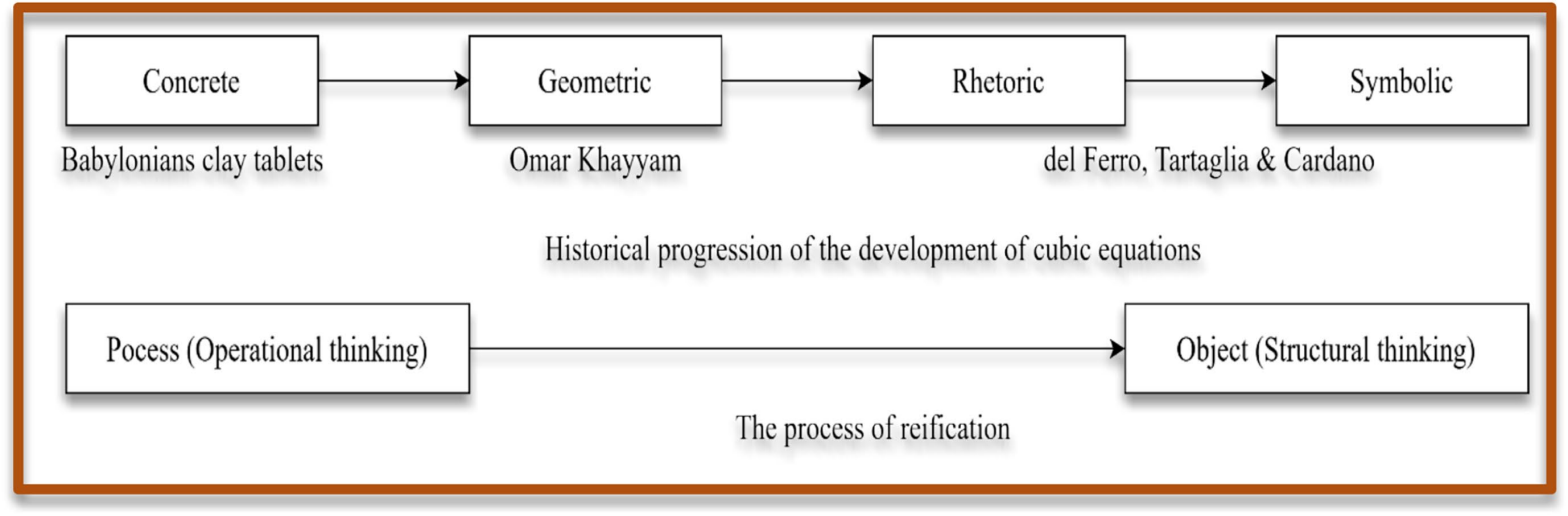
Aligning the history of cubic equations with the process of reification.

Focus in this paper specifically has been given to cubic equations and how its history can be exploited for this advantage. In this paper, we follow that the history of cubic equations may provide a natural context for reification process according to Sfard’s theory of reification. In this view, students transition from operational thinking to structural thinking when learning algebraic concepts (Sfard, 1995). In the operational thinking, students focus on how to perform operations, such performing step-by-step procedures to solve equations or manipulate expressions (Sfard and Linchevski, 1994). In contrast, structural thinking emphasises what the mathematics object represents, its inherent properties, and its relationships within a broader system (Kieran and Sfard, 1999; Zerpa, 2016), which enable students to reason flexibly and make connections across different algebraic representations (Brenner et al., 1997; Newton et al., 2010). This transition is more aligned with the historical progression of the concept of cubic equations (see Figure 2). Earlier, the Babylonians employed concrete means for solving problems that involved finding cube roots (Høyrup, 2019; Katz, 2021). This reflects an operational or procedural conception of the concept. In the early Islamic period, mathematicians such as Omar Khayyam solved specific types of cubic equations using geometric constructions and rhetoric formulations (Rashed, 2013). His approaches reveal a deep structural awareness, though expressed in non-symbolic forms. During the Italian Renaissance, del Ferro, Tartaglia, and Cardano revolutionised solution algorithms by developing general symbolic methods for solving cubic equations. This development from rhetoric to symbolic representations and generalisations allows procedures to be mentally internalised as processes (Dubinsky and McDonald, 2001). Finally, algebra emerged and the formalisation of functions and equations as abstract objects enabled a structural understanding (Corry, 2003), where concepts could be manipulated, analysed, and related systematically (Dubinsky and McDonald, 2001).

Viewing the history of cubic equations at a pedagogical standpoint in line with the reification process, teachers can scaffold a similar cognitive trajectory using this history to help students move from empirical to structural reasoning. The process of transforming and “depressing” cubic equations to simplify their structure exemplifies reasoning about invariance and transformation, two of the important aspects of structural thinking (Harel, 2018). Thus, studying the history of the cubics provides a natural context for helping students perceive algebraic relationships as structured systems rather than isolated procedures.

## Method

### Design

**Figure 3:**
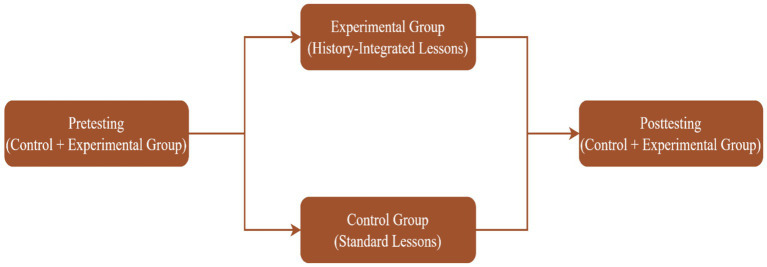
Study design.

This study employed a quasi-experimental non-equivalent control group design to examine the effectiveness of the history integrated teaching intervention on students’ structural reasoning in cubic equations in a senior high school setting. In this study, quasi-experimental design is appropriate since random assignment is not feasible due to natural classroom groupings (Shadish et al., 2002; Gall et al., 2007). Intact classes were used as the experiment and control groups. Two intact Form 2 classes participated in the study. In order to mitigate cross-group effect, the classes were taken from two different senior high schools in the Ashanti region of Ghana. The experiment group class received the historical teaching intervention while the control group class were taught with the standard instruction contents prescribed in the curriculum. Although the groups were not randomly assigned, efforts were made to ensure that the grouping is homogenous and hence more comparable. This was achieved by selecting classes taught within the same level (Form 2), and following the same further mathematics contents (Figure 3).

### Participants

In Ghana, senior high school education typically spans 3 years (Forms 1–3) and follows 9 years of basic education, which includes 6 years of primary school education and 3 years of junior high school education. Mathematics instruction at the senior high level is primarily in English, and students must take the West African Senior School Certificate Examination (WASSCE), pass mathematics before they are said to qualify and be eligible for tertiary education. Learning is exam-oriented, and this has culminated in students focusing on procedural understanding than deeper algebraic understanding. The participants recruited for this study were second-year (Form 2) senior high school science students who were enrolled in Mathematics (Elective). These students were drawn from two intact science classes located in two different public senior high schools within the Ashanti Region of Ghana. The sample therefore reflected naturally occurring classroom groups rather than artificially assembled cohorts. This ensured that the participants’ learning experiences and interactions remained authentic to their usual instructional environments. In total, the study involved 102 male and 26 female students. Participants age ranged between 15 and 17 years old with average age of approximately 16 years old. A convenience sampling technique was employed to select both the schools as the study sites and the individual student participants to make each group (control or experimental). Primarily due to the accessibility of the selected schools and the willingness of the students to participate, convenience sampling was seen appropriate. Moreover, convenience sampling was deemed appropriate for an exploratory educational intervention, where practical considerations and classroom dynamics play essential roles in the feasibility of implementation. After the post-test data collection and analysis, 10 students were purposefully selected from the experimental group. This number of students was selected based on the principles of theoretical data saturation which occurred on the 11th interviewee (Guest et al., 2006). These students were individually interviewed to provide further explanations to why and how there was enhanced students structural reasoning. Student participation was entirely voluntary, and each student was informed about the purpose and procedures of the study prior to data collection. Only students who provided consent were included.

### Lessons design

Both the experimental and control groups were taught the same topic of cubic equations. The instructions were structured over four lessons. Each lesson lasted for 60 min. So, in all, each group was taught over a total instructional time of 240 min, that is, 60 min per lesson. Despite the fact that the content coverage was identical across both groups, the instructional design of each lesson differed significantly. In all the groups, the lessons conclude with assessment tasks (structural reasoning tests).

#### Experimental group

The lessons that the experimental group was taught were designed taking antecedents of the history of cubic equations into consideration. The design of the lessons emphasised how early mathematicians thought about and dealt with the properties that helped mathematicians to deduce the structures inherent in cubic equations.

##### Lesson 1: recognising structure in cubics

The teacher introduced this lesson by outlining the historical background related to how ancient mathematicians viewed the concept when there was no symbolic algebra. Omar Khayyam, for example, viewed a cubic equation as a relationship between three geometric magnitudes: a cube (volume), a square or rectangle (area), and a line (length). So, according to his book titled the *Treatise on Demonstration of Problems of Algebra* (c. 1,070) (see Siadat and Tholen, 2020), in Khayyam’s view, a cubic equation is a geometric equality involving volumes, areas, and lengths. From this historical idea, the teacher explained the algebraic conceptualisationof cubic equations as “polynomial equations of the third degree”, giving the general form as $ax^{3}+bx^{2}+\mathrm{cx}+d=0$ and examples like $x^{3}+2x^{2}-5x-6=0$, taking into account the historical background that lies during the Renaissance when rhetoric algebra existed (see Toscano, 2020). Then, he asked the students in the class to sort 10 given equations into quadratic and cubic forms. They discussed the grounds upon which the sorting was done. Students were then guided to work in small groups to classify cubic equations into pure cubics (e.g., $x^{3}-8=0$), cubics with missing terms (e.g., $ax^{3}+\mathrm{bx}+c=0$), and general cubics (e.g., $ax^{3}+bx^{2}+\mathrm{cx}+d=0$) (Kalantari and Zaare-Nahandi, 2022).

All these classifications were based on the structural identifications found in the history of the concept (Deakin, 2005; Connor, 1956; Guilbeau, 1930).

##### Lesson 2: Khayyam’s classification

In this lesson, the teacher introduced the different classifications of cubic equations offered by Omar Khayyam during the medieval ages. Khayyam classified cubic equations into 13 distinct types and solved them geometrically by drawing conic sections and using the intersections. He did this because that time symbolic algebra was not known (Kent and Muraki, 2016; Siadat and Tholen, 2020; Siadat and Tholen, 2020). Using this historical background, the teacher presented different forms of cubic equations (e.g., $x^{3}=\mathrm{ax}+b$) and explained how Khayyam went about dealing with them using geometry despite the absence of symbolic algebra. From here, theteacher guides students to work collaboratively to match modern cubic equations to these historical types and justify their classifications. Discussions focused on why classification was based on cubic structures and how it informed solution strategies (Kalantari and Zaare-Nahandi, 2022).

The teacher introduced the challenges that lies when mathematicians tried to solve cubic equations in the olden days, which prompted them to explore these structures, looked into the characteristics within the structures that remain the same when they compared different forms of cubic equations (that is, invariants), and exploit these structures to help ease the difficulty involved in solving cubic equations (Kalantari and Zaare-Nahandi, 2022).

##### Lesson 3: depressing the cubic

The teacher explained the reasons behind why it was important to transform algebraic structure as it was informed in the history of cubic equations. The teacher introduced the historical concept behind the idea of transforming a general cubic into a depressed cubic. This idea emerged in the work of Tartaglia and, later published by Cardano in the Ars Magna (1545) during the Renaissance period (Kouropoulos, 2025). The main strategy rests on transformations to simplified cubic of the form, for example, $x^{3}+\mathrm{bx}=c$. The aim was to remove the *x*^2^ term that maintains the difficulty in solving the general cubic or cubics that has this term. When theis term is removed, the final equation is called the ‘depressed cubic’ (Strobach, 2016). To depress a cubic, ancient mathematicians used the substitution, $x=y-\frac{b}{3a}$. A worked example of depressing the cubic equation, $x^{3}-9x^{2}+27x-27=0$ was used to demonstrate how the substitution $x=y-\frac{b}{3a}$ works. In this case, the substitution that was made is x=y+3 and the final depressed cubic equations was $y^{3}=0$.

Students then transformed given cubic equations into the form $y^{3}+\mathrm{py}+q=0$. They identified invariants such as degree, number of roots, and general graph shape, and discussed why these properties remain unchanged even under transformation.

##### Lesson 4: graph behaviour

Students analysed different forms of cubic equations (e.g., pure, symmetric, and general). Working in groups, they predicted: the number of roots, turning points, symmetry, and end behaviour by using the historical classifications by, for example, Khyyam (Eves, 1958).

Students justified their predictions by thinking about the cubic structures rather than direct calculations or graphing. The lesson concluded with students transforming cubic equations, sketching their general shapes, and annotating key invariants by giving them exercises and take-home assignments.

#### Control group

The control group lessons followed the standard instruction that focused on guiding students develop the ability to apply procedures. This approach is well supported by the instrumental understanding view of mathematics teaching and learning (Skemp, 1976; Star, 2020). For the sake of comparison, the lessons covered the same contents as that of the experimental group but they were taught without any inclusion of historical context. The breakdown of the lessons is as follows:

##### Lesson 1: introduction to cubic equations

The teacher defined cubic equations and introduced the general form $ax^{3}+bx^{2}+\mathrm{cx}+d=0$. Students were shown how to identify coefficients and missing terms.

Worked examples were provided, followed by individual exercises in classifying equations.

##### Lesson 2: forms and classification of Cubics

The teacher presented different forms of cubic equations and demonstrated how to rewrite equations into the standard form.

Students practiced classification through routine exercises. The focus was on correct identification using rules, with minimal discussion.

##### Lesson 3: transformation to depressed form

The teacher introduced the substitution $x=y-\frac{b}{3a}$ as a standard method. A step-by-step procedure was demonstrated.

Students applied the method to several examples individually. The emphasis was on accurate execution of the procedure, rather going deeper into how it came about or the conceptual knowledge behind it.

##### Lesson 4: graph behaviour

The teacher explained key features of cubic graphs, including intercepts, turning points, and end behaviour.

Students observed worked examples and practiced sketching graphs based on given equations. Graph interpretation relied on rules and demonstrated examples.

### Procedures

**Figure 4:**
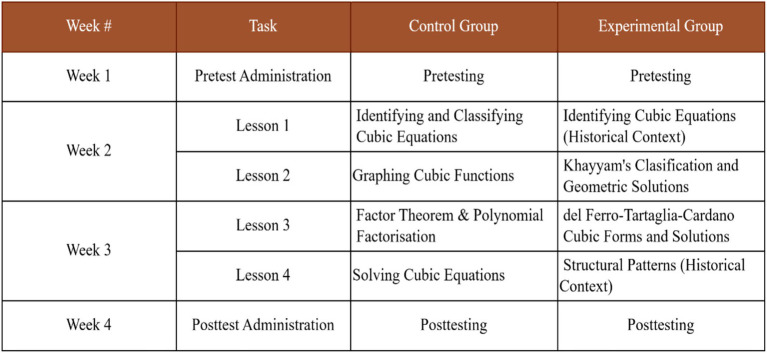
Research procedures.

Figure 4 illustrates the sequence of the procedures adopted for this study. It shows that the research process took 4 weeks. In the first week (week 1), both the control and experiment groups completed a pretest on structural reasoning test prior to the implementation of the teaching intervention. This was done to strengthen internal validity of the study; it allowed for establishing baseline equivalence among the participants in both groups (Shadish et al., 2002). It also helped to test the reliability of the instrument and made any changes to the items or their phrasing to enhance the reliability (Hilton, 2017). After the pretesting, the teaching intervention was commenced in week 2. The lessons which integrate historical sources of cubic equations was taught among the experiment group over 4 lessons following a 2-week-long teaching plan, where the researcher met the class four times in week 2 and week 3 with each lesson taking 60 min in duration per day (see Figure 4 for the breakdown). In the same 2 weeks, as the experimental group is taught the history-integrated lessons, the control group on the other hand were receiving standard instruction on cubic equations as prescribed by the further mathematics curriculum and teachers manual. The control group also met the researcher 4 times (4 lessons) in the week 2 and week 3 with each lesson taking 60 min per day. What changes in the lessons content is the historical sources used on the experimental groups. Other than that, all the groups were taught the same concept of cubic equations, which confirm that there is no bias in terms of content selection. In all, each of the groups has been met for 240 min (4 h) for the 2 weeks. Following the intervention, both groups completed post-tests in week 3. The post-test was designed to measure improvements in structural reasoning after the teaching intervention. Throughout the study, care was taken to maintain among all groups consistent instructional time, testing conditions, and instructional content (focus on cubic equations) across the two groups to further reduce potential confounding variables. This research procedure provided a feasible and methodologically sound approach for testing the historical intervention within an authentic senior high school context.

### Instrument

The instrument for this study is an objective test with options lettered A—D. Students are instructed to circle the best option that explains the situation described in each question about the structure of cubic equations. The test was designed to measure students reasoning about cubic structures according to five dimensions of the conceptual framework of structural reasoning offered by Harel and Soto (2017). Thus, the test comprised of five (5) sections. Each section has a total of 6 questions asked to measure particular dimension of structural reasoning (see Figure 1) in cubic equations. So, in all the test comprised of 30 objective test items. The description of the test with example items and answers (in brackets) under each dimension are given as follows.

The first section made up items to measure students’ “pattern generalisation” ability in cubic equations. Examples of items include: (i) *which feature distinguishes cubic equations from quadratic equations? (3rd degree)* (ii) *why can a cubic equation have one or three real roots but not exactly two (its graph crosses the x-axis three times or once)*. The second section consisted of items to measure students’ ability to “reasoning in terms of general structure” of cubic equations. Two of the items here were as follows: (i) *If all the coefficients in* $x^{3}+bx^{2}+\mathrm{cx}+d=0$ *are doubled, what happens to its graph? (it becomes steeper)* (ii) *If* ${\left(x-a\right)}^{3}=0$*, then its inflection point is …….* (a,0). The third section comprised of items that were used to asses students’ ability to “reduce an unfamiliar cubic structure to familiar one” so that working with the new structure becomes easier. For example, students were asked these two questions: (i) *The cubic equation* $3x^{3}+24$ *can be reduced by ……. (sum of cubes),* (ii) *To solve* $x^{3}+bx^{2}+\mathrm{cx}+d=0$*, we use the substitution …….* (x=y−b/3). The fourth part involved items designed to measure students’ ability to “reason & operate with structure in thought”. The following items, for example, were used to measure this dimension of structural thinking: (i) *From the structure of* ${\left(x+2\right)}^{3}=0$*, you can predict ……. (root)*, (ii) *To show that* x=−2 *is a root of* $x^{3}+6x^{2}+12x+8=0$*, we rely on ……. (factor-root connection)*. Finally, there were also items constructed to measure students “epistemological justification” in cubic equations. These items include, for example: (i) *why do we have to set* x=u+v*, when solving cubic equations of the form* $x^{3}+\mathrm{ax}=b$ *(to remove the*ax *term),* (ii) *why do we have to remove the* $x^{2}$ *term when solving cubic equations of the form* $x^{3}+bx^{2}+\mathrm{cx}+d=0$ *(to make the equation symmetric for solving)*.

The test scripts of the two groups were collected and scored independently by two raters under the supervision of the first author. One rater worked on the scripts of the control group and the other worked on that of the experimental group. The raters were master of philosophy students specifically trained by the first author to do this job. The rating followed that, if a student circle the correct answer, she/he is awarded 1 point, otherwise 0 point. Given that a student gets all answers correct, the maximum number of points in all would be 30. Raters made these decisions based on a marking scheme or scoring rubric developed by the first author. Scores were coded into SPSS (version 27) and subjected to reliability analysis using Kuder–Richardson Formula 20 (KR-20) which is specifically designed to measure the internal consistency for dichotomous items. Results showed KR-20 coefficient of 0.86. This demonstrated high level of internal consistency among the objective test items (Nunnally and Bernstein, 1994) and it confirmed that the test consistently measured the intended construct.

In addition to the test designed to collect quantitative data, a semi-structured interview guide was developed to be used to gather qualitative data to help explain and enrich the results obtained from the analysis of the quantitative data. The interview guide was used on selected students in the experimental group. On the interview guide, two questions were asked. These questions are: (i) *How did learning cubic equations through their historical development help you recognise patterns and relationships between the terms in the equations?* (ii) *Can you describe how your approach to solving cubic equations changed after participating in the history-integrated lessons? How do you now think about the structure of these equations?* The first question was asked to probe how students understood structural features that describe the behaviour of cubic equations such as coefficients, missing terms, and classification of cubic forms. The second question was also asked to explore how students could articulate structural reasoning, for example, recognising patterns, linking transformations, or understanding coefficient-root connection, etc.

### Data analysis

Analysis of the pretest data using independent sample t-test revealed that: (i) the variances of the two groups were homogeneous [F(126)=0.926,p=0.338], but (ii) there was statistically significant difference between the two groups in terms of initial structural reasoning ability scores in favour of the control group (t=−2.525,df=126,p=0.013). Given this pre-existing difference and considering that the post-test data was not normally distributed revealed through Kolmogorov–Smirnov test [D(n=128)=0.100,p=0.003], it was deemed appropriate to employ non-parametric analysis of covariance (ANCOVA), in this case, the Quade’s test for the final data analysis. Quade’s test is data analysis technique that is robust for adjusting post-test data for pretest (baseline) differences in the case where data is not distributed normally. This helps to provide more valid estimate of the effect of an intervention (Quade, 1984). The choice of Quade’s test (non-parametric ANCOVA) is appropriate since there was the need to control for the difference in the pretest and accurately assess the effect of the history-integrated lessons on students structural reasoning, despite the fact that the data were not normally distributed (Carlsson et al., 2014).

In addition to the quantitative analysis, qualitative data were analysed using thematic analysis. Audio-recorded interviews were transcribed verbatim on participant-by-participant basis. The analysis followed the six steps of reflexive thematic analysis offered by Braun and Clarke (2006, 2021, 2023). This process begun with (i) repeatedly reading the transcripts to become immersed in the data to understand the contents, (ii) inductively generating codes to identify meaningful features of responses, (iii) organising codes into potential themes which represent recurring patterns in the data put under each heading, (iv) review and refine themes to ensure that they are coherent, internally consistent and accurately represent the data, (v) clearly defining each theme and supporting it with relevant illustrative quotations, and ends with (vi) putting together the themes and writing the results into a report. In fact, the purpose of doing this was to help answer questions as to why and how the results obtained in the quantitative analysis were so.

## Results

### Improvements in structural reasoning

To find out whether there were higher improvements in structural reasoning scores among the students in the experimental group than the students in the control groups, Quade’s test was computed in SPSS (version 27) on the post-test data using the pretest data as covariate. This was done to adjust for the initial difference in structural reasoning scores as it was identified from the analysis of the pretest data. Table 1 shows results of intervention effects obtained from the Quade’s test output.

**Table 1:** Quade’s test results for post-test structural reasoning scores adjusted for pretest differences.

	Sum of squares	df	Mean square	*F*	*p*-value
Between groups	21,748.6306	1	21,748.6306	18.050	<0.001
Within groups	151,818.2153	126	1,204.9065		
Total	173,566.8459	127			

According to Table 1, the results revealed a statistically significant difference between the experimental and control groups on post-test structural reasoning scores [F(126)=18.050,p<0.001]. This shows that students in the experimental group who received the history-integrated lessons on cubic equations demonstrated higher scores in structural reasoning than their counterparts in the control group who did not receive history-integrated lessons. The history-integrated lessons had a large effect size on structural reasoning scores ($\eta^{2}=0.125$). Partial eta squared of this value indicates that approximately 12.5% of the proportion of variance in post-test scores was attributable to the intervention after adjusting for pretest differences. Many researchers consider eta squared above 0.06 to indicate above medium to large effect which could be practically significant in the area of educational research (e.g., Richardson, 2011). Based on these results, we accepted the alternate hypothesis and conclude that there exists significant statistical difference in terms of structural reasoning between students who participated in the history-integrated lessons and those that did not.

### Learning experiences and reasoning processes

To explain how students in the experimental group after participating in the history-integrated lessons helped them perform higher in the structural reasoning test, we conducted thematic analysis of the interviewed data. We did this by following the six-step procedures offered by Braun and Clarke (2006). Table 2 shows that we identified three main themes that emerged from the analysis of the data. These themes were (1) enhanced recognition of structural features, (2) deeper conceptual understanding of cubic equations, and (3) shift in equation-solving thinking. These themes capture how students reflected on structural properties of cubic equation forms after they have participated in learning cubic equations through its history.

**Table 2:** Themes.

Theme	Codes	Illustrative quotes
Enhanced Recognition of Structural Features	Identification of coefficients and missing terms	‘Learning how cubic equations were developed by mathematicians in history really helped me see patterns in the equations. I noticed that when some terms are missing, like the linear or constant term, it changes how I can solve it.” (S1) “I discovered that equations follow certain patterns. For example, missing terms show special forms and the coefficients tell me how the roots behave.” (S3)
	Classification of cubic forms	“The history lessons made me understand why there are different types of cubic equations. I can now see how the numbers in front of *x*^3^, *x*^2^, and *x* affect the solutions.” (S2) “The historical examples helped me see cubic equations as organised. I can now tell how each term affects the roots and the graph.” (S10)
Change in Equation-Solving Thinking	From procedural to structural thinking	“Before, I used to just apply formulas blindly. Now I first check the equation’s structure and missing terms to know the best way to approach it.” (S1) “I now take my time to look at the equation and classify it before trying to solve. I think about the structure first, not just the answer.” (S2)
	Strategic examination of structure	“I now examine the equation’s form first and predict the kind of solution I might get.” (S3) “I now plan my solution around the equation’s structure instead of trying random formulas.” (S6)
Deeper Conceptual Understanding	Interconnectedness of features and solutions	“I noticed that the same forms of cubic equations appear again and again. Studying history helped me recognise these patterns.” (S8) “The historical perspective helped me see cubic equations as organised systems.” (S10)
	Meaningful engagement with equations	“I now think carefully about the structure before solving. It makes the work easier and less confusing.” (S8) “My approach is more thoughtful now. I focus on understanding the structure and relationships before solving.” (S10)

#### Enhanced recognition of structural features

Students reported that when they learned about the history of the development of cubic equations, it helped them to identify the structural patterns inherent in different cubic forms. They said they were able to see how coefficients and missing terms influence solving the equations. For example, one student, S1 said *“learning how cubic equations were discovered by old mathematicians in history really helped me see patterns in the equations. I noticed that when some terms are missing, like the linear or constant term, it changes how I can solve it”*. Another student emphasised the classifications of cubic forms offered in the history. S2 in his experience said that “*The history lessons made me understand why there are different types of cubic equations. I can now see how the number in front of x^3^, x^2^ and x affect the solutions”*. These responses indicated that when historical pieces were introduced into lessons, it enhanced students’ ability to recognise and interpret the features that make up the structure of cubic equations. This includes understanding missing terms and how coefficients relate to one another, all these important to understanding cubic equations.

These suggest that the historical context helped students promote their thinking from viewing cubic equations as abstract to recognising that they have systematic structural forms that makes them unique. This recognition likely contributed to improved strategic reasoning, as students could anticipate solution strategies based on equation structure. Moreover, the responses on missing terms and coefficients indicates that students internalised the concept of invariants, which are structural elements that remain constant under different forms of cubic equations. As it is noted, knowledge of invariants enhanced participants ability to reason about cubic equations beyond procedural application alone. The history integrated lessons helped them identify structural patterns, such as missing terms and coefficients, which may guide their approach to problem-solving. Across participants, recognising these structural elements became a strategic heuristic, which helped them to anticipate solution methods. This highlights how history-integrated lessons can support the internalisation of mathematical structure, where it improves students’ ability to reason structurally rather than procedurally.

#### Deeper conceptual understanding

Secondly, students reported a more holistic understanding of cubic equations. They recognised that they got to know better how coefficients, terms, and roots of cubic equations connect to each other. Through this knowledge they revealed appreciation of the logical structure underlying cubic equations. S10 reported that *“The historical examples helped me see cubic equations as organised structure. I can now tell how each coefficient of the terms can affect the roots and the graph”*. S8 also indicated *“I noticed that the same forms of cubic equations appear again and again. Studying history helped me recognise these patterns.”* Students also reported that learning through the history helped them see learning about the concept of cubic equations more meaningful and less intimidating. This could have contributed to the deeper conceptual understanding that students recalled. S8 noted *“I now think carefully about the structure before solving. It makes the work easier and less confusing”*. S10 added that *“My approach is more thoughtful now. I focus on understanding the structure and relationships before solving.”* These responses indicated that when students are engaged in history-integrated lessons, they improved on their ability to reason profoundly about concept where students develop enhanced confidence in working with cubic equations.

These indicate that history-integrated lessons promoted conceptual understanding rather than procedural understanding. Students were not merely thinking of applying memorised formulae; instead, they can recognise recurring structural patterns and relationships between the characteristics of cubic equations. This deeper conceptual grasp is closely aligned with enhanced in structural reasoning, as students could predict and analyse solutions based on an understanding of underlying mathematical structures. The recurring emphasis on pattern recognition also suggests that students developed meta-cognitive awareness about their problem-solving processes, a skill that extends beyond thinking about individual equations.

#### Change in equation-solving thinking

The third theme identified from the students’ responses shows a transition from procedural to analytical thinking when solving equations. Prior to the history-integrated lessons, students often relied on memorised algorithms such as the rational-root test but after the history-integrated lessons, they reported that before they solve any given cubic equation, they had to examine its structure. For example, S1 recalled that *“Before, I used to apply formulae just like that. Now I first check the equation’s structure and missing terms to know the best way to approach it.”* S2 also noted that *“I now take my time to look at the equation and classify it before trying to solve. I think about the structure first, not just the answer.”* Students also described that before they begin to solve any given equation, they strategically have to analyse the structure to decide which method could be more appropriate. S3 reflected that *“I now examine the equation’s form first and predict the kind of solution method I might get.”* S6 reported *“I now plan my solution around the equation’s structure instead of trying any formulae.”* These insights suggest that students developed structural reasoning, allowing them to make informed decisions and approach cubic equations with greater conceptual understanding.

This theme emerged from responses that highlight how historical context encouraged students to develop strategic reasoning skills. This aligns with structural thinking. Students’ reflections show that they were actively evaluating the features of equations, such as missing terms, coefficient patterns, and form classification, before selecting a solution method. This strategic approach demonstrates that students were internalising structural reasoning heuristics, which likely explains their higher performance on the structural reasoning test. Importantly, the consistency across multiple participants suggests that the historical lessons fostered transferable problem-solving strategies, rather than isolated improvements on individual tasks.

## Discussion

The purpose of this study was to investigate whether integrating elements of the history of mathematics concepts could help improve on students algebraic thinking. Specifically, the study focused on how using the history behind the development of the concept of cubic equations could enhance senior high school students’ structural reasoning ability about the concept. The findings provide robust quantitative and qualitative evidence that converge to support that when history of concepts is integrated into mathematics lessons, it can meaningfully support students’ ability to reason about algebraic structure inherent in cubic equation forms. The results are interpreted and discussed in relation to the theoretical framework of structural reasoning (e.g., Harel and Soto, 2017), theories of algebraic cognition, especially those that talks about reification process (Sfard, 1991, 1995; Kieran, 2006; Duval, 2006), and the literature on the pedagogical use of the HoM (e.g., Fried, 2001, 2018; Katz and Barton, 2007; Radford, 2014).

There is the finding that there exists statistically significant difference in post-test structural reasoning scores between the experimental and control groups. This finding indicates that when students learned cubic equations following the historical progression of the development of the concept, the lessons were effective in promoting deeper thinking about the algebraic structures inherent in cubic equations. This finding directly supports the findings of the empirical studies that reports the positive effects of using the history of mathematics on students understanding of algebraic concepts (e.g., Florio, 2020; Heeffer, 2007; Nataraj and Thomas, 2016). While students in the experimental groups merited from the history-integrated lessons, students in the control group on the other hand received standard lessons more inspired by the mathematics curriculum. The finding goes with the interpretation that such lessons are always designed geared to promote the procedural knowledge of students in executing step by step algebraic operations at the expense of making good meaning from algebraic representations. This could be the reason why the group did not exhibit comparable growth in structural reasoning. This reinforces the idea that when teachers focus on teaching lessons that aim at procedural proficiency alone, it does not guarantee structural understanding (Sfard, 1991; Susac et al., 2014).

The findings of the effectiveness of the history-integrated lessons can be interpreted in the perspective of the history and epistemology of mathematics (HPM). The HPM emphasises that mathematics exists both as body of knowledge and a process of knowledge creation (Clark et al., 2016). In relation to the present findings, the HPM dimension of seeing mathematics as a process of knowledge creation comes in to explain it in such a way that, engaging students with the epistemological struggles and conceptual breakthroughs surrounding cubic equations transforms how they see and understand algebra concepts. Students begin to change from seeing algebra concepts as static set of rules that they should consume in order to do well in mathematics into seeing it as an evolving system of mathematics ideas that are defined by some structures. This aligns with the argument of Fried (2014) that integrating historical pieces into instruction can act as a pedagogical bridge between students’ cognitive development and the epistemological structure of mathematics.

The concept of structural reasoning is conceptualised based on five dimensions in the present study (Harel and Soto, 2017). So, if there is improvement in structural reasoning, that improvement also cuts across all the five dimensions that make up the conceptualisation. One important outcome of the history-integrated lessons was students’ improved ability to recognise and generalise patterns within cubic equations. Interview responses indicated that students became more aware of how coefficients, missing terms, and equation forms relate to the number and nature of roots. This finding aligns with Harel and Soto (2017) distinction between result-pattern and process-pattern generalisation. Rather than merely observing that cubic equations often have one or three real roots, students began to reason about *why* these patterns occur, linking algebraic form to graphical behaviour and underlying structure of solutions.

The history behind the development of cubic equations naturally foregrounds such generalisations. Early mathematicians did not come out with a single universal formula or algorithm to solve the equations but instead classified cubic equations into distinct forms, each requiring different solution strategies or formula (Rashed, 2013; Katz, 2021). Students were exposed to algebraic generalisation as a human activity rooted in recognising structural regularities by retracing this classificatory process. This supports the assertion that historical reconstructions can foster semiotic and conceptual development by making patterns explicit and meaningful (Radford, 2014).

Another dimension of structural reasoning strengthened by the intervention was students’ ability to reduce unfamiliar structures to familiar ones. As theorised by Hoch and Dreyfus (2006) and Linchevski and Livneh (1999), structure sense involves recognising when an algebraic expression can be transformed into an equivalent but more tractable form. Students’ increased understanding of transformations such as depressing the cubic illustrates this ability. From a theoretical perspective, this result resonates with reification process (Sfard, 1995). Historically, the transformation of the general cubic equation into a depressed form represented a major conceptual advance. It helped mathematicians to focus on invariant structure rather than how complex cubic forms are when looked at from surface. When students engaged with this transformation that has historical significance, they were not merely following procedures to solving equations but they were developing an understanding of why certain forms of algebraic representations are mathematically advantageous. This supports the idea of Duval (2006) that conceptual understanding in algebra depends on coordinating transformations across representations while preserving structural meaning.

Findings from the analysis of the interview data further revealed a clear change in students’ cubic equation-solving understanding and how they might use approaches, from just applying memorised algorithms to strategically analysing structure before solving. This change is well aligned with the reification process of algebraic cognition that emphasise the transition from operational to structural conceptions (Sfard, 1991; Kieran and Sfard, 1999). Prior to the lessons, students often relied on procedures such as the rational root test without attending to the internal organisation of the cubic equations. After the lessons, students reported having to closely examine coefficients, missing terms of cubic equations, and symmetry before choosing a solution method that fits appropriately. This result aligns with the notion of process conception (Dubinsky, 1991) which aligns with the APOS theory (Dubinsky and McDonald, 2001). It emphasises the mental manipulation of algebraic structures. By learning how historically mathematicians reasoned about cubic equations, students began to operate on algebraic forms in thought, predicting roots and solution strategies without extensive symbolic manipulation. Such reasoning reflects what Harel (2013) describes as acting upon structures rather than merely executing procedures.

The findings also indicate that students developed an improved ability to reason in terms of general cubic structure. Students’ reflections suggest that they began to see individual cubic equations as instances of a broader conceptual entity, characterised by invariant properties such as degree, inflection point, and coefficient–root relationships. This aligns with Tall and Vinner (1981) notion of concept image and with Dorier and Sierpinska’s (2001) emphasis on recognising similarities and reorganising knowledge. The historical trajectory of cubic equations from specific rhetorics to general symbolism mirrors this cognitive progression. As Radford (2001, 2014) argues, algebraic generality emerges through the gradual abstraction of structure from concrete problem situations. By reenacting this progression pedagogically, the intervention supported students’ ability to reason beyond specific examples and engage with cubic equations as general mathematical objects.

This study extends the literature in several important ways. First, it provides empirical evidence that HoM can support structural reasoning, a learning outcome that has received less attention than conceptual or procedural understanding in prior HoM studies. Second, it addresses an underexplored instructional context: cubic equations at the senior high school level. Much of the existing research on historical integration focuses on elementary or lower-secondary topics (e.g., square roots, logarithms), leaving higher-degree polynomials largely unexplored. Finally, by explicitly grounding the intervention in the structural reasoning framework of Harel and Soto (2017), this study strengthens the theoretical connection between historical pedagogy and algebraic cognition. The findings suggest that history is not merely an enrichment tool, but a powerful pedagogical resource for fostering advanced forms of algebraic thinking.

## Implications for research and practice in mathematics education

The findings of this study suggest that integrating elements of the history of mathematics concepts into algebra instruction can serve as an effective means of fostering structural reasoning, particularly in advanced topics such as cubic equations. Rather than treating history as an add-on or enrichment activity to standard instruction, teachers can use historical antecedents behind the development of concepts as a central instructional resource to highlight why algebraic structures take the forms they do and how solution methods emerged in response to conceptual challenges. Such an approach encourages students to analyse equations strategically, examine their structure before selecting solution methods, and reason about general properties rather than relying solely on memorised algorithms. In exam-oriented educational contexts, where procedural competence often dominates classroom teaching, history-integrated instruction offers a way to balance procedural fluency with conceptual and structural understanding. For teachers, this implies they could change their approaches from presenting algebraic techniques as finished products towards framing them as solutions to conceptual problems. Teachers may, for example, explicitly connect historical transformations, such as depressing the cubic, to modern solution strategies, thereby helping students to see algebraic manipulation as purposeful and meaningful. At the curriculum level, the results support calls for greater emphasis on reasoning about structure as a core algebraic competence. Curriculum developers may consider embedding historically informed tasks and narratives within algebra units, especially those dealing with complex symbolic forms. Such integration can help make abstract content more accessible and intellectually coherent. This study underlines the value of explicitly targeting structural reasoning as a learning outcome in algebra research. It demonstrates that historically informed instructional designs can be systematically linked to cognitive frameworks such as that of Harel and Soto (2017). It then offers a productive avenue for theory-driven intervention research. The findings invite further exploration of how history of different mathematics concepts and instructional designs may support specific dimensions of algebraic reasoning.

## Limitations and suggestions for future research

Despite contributions made by the study, it has several limitations that should be acknowledged. First, the use of a quasi-experimental design with intact classes limits the extent to which causal inferences can be generalised beyond the study context. Although statistical controls were applied to address pre-existing differences, future studies employing randomised controlled designs would strengthen claims about the causal impact of history-integrated instruction. Second, the sample was drawn from two senior high schools within a single region of Ghana, which may limit the generalisability of the findings to other educational contexts beyond the region, curricula, or cultural settings. Replication studies across different regions, school types, and national contexts would help establish the robustness of the observed effects. Third, the intervention focused specifically on cubic equations over a relatively short instructional period. While this focus allowed for depth, future research could investigate whether similar effects occur with other advanced algebraic topics, such as quartic equations, polynomial functions more broadly, or abstract algebraic structures. Longitudinal studies would also be valuable in examining whether improvements in structural reasoning could be sustained over time and could be transferred to new mathematics domains. Finally, although this study incorporated qualitative interviews to illuminate students’ experiences, further research could employ more fine-grained qualitative methods, such as classroom observations or task-based interviews, to capture how structural reasoning develops moment by moment during history-integrated instruction. Such study would deepen understanding of the mechanisms through which historical contexts support algebraic thinking. In sum, while the present study provides strong evidence for the effectiveness of integrating the history of cubic equations to enhance structural reasoning, it also opens multiple avenues for future inquiry aimed at strengthening the theoretical, empirical, and practical foundations of history of mathematics informed mathematics education.

## Data Availability

The raw data supporting the conclusions of this article will be made available by the authors, without undue reservation.

## References

[ref1] Apawu J. Akosua N. Ansah O. Akayuure P. (2018). A study on the algebraic working processes of senior high school students in Ghana. Eur. J. Sci. Math. Educ. 6, 62–68. doi: 10.30935/scimath/9523

[ref2] Arthur Y. D. Appiah S. K. Amo-Asante K. Asare B. (2022). Modeling student’s interest in mathematics: role of history of mathematics, peer-assisted learning, and student’s perception. Eurasia J. Math. Sci. Technol. Educ. 18, 1–10. doi: 10.29333/ejmste/12458

[ref3] Arthur Y. D. De Vittori T. Welcome N. B. De Vittori T. Dogbe C. S. K. Asare B. (2025). Enhancing TVET students’ interest in mathematics through the history of mathematics and self-efficacy. J. Appl. Res. High. Educ. 18. doi: 10.1108/JARHE-05-2024-0238

[ref9001] Association Center for Best Practices and Council of Chief State School Officers. (2010). Common core state standards for mathematics. Washington, DC: Authors.

[ref4] Baki A. Guven B. (2009). Khayyam with Cabri: experiences of pre-service mathematics teachers with Khayyam's solution of cubic equations in dynamic geometry environment. Teach. Math. Its Appl. 28, 1–9. doi: 10.1093/teamat/hrp001

[ref5] Bannor G. A. Arthur Y. D. Obeng B. A. (2024). Effects of perceived mathematics connection on mathematics motivation: mediating role of history of mathematics concepts. Int. J. Math. Math. Educ. 2, 1–14. doi: 10.56855/ijmme.v2i1.898

[ref6] Barbin É. (2013). “History and pedagogy of mathematics in France,” in History of Mathematics, in Encyclopedia of Life Support Systems. Dordrecht: Springer.

[ref9002] Barbin É. Tzanakis C. (2014). History of mathematics and education. In Lerman S. (Ed.), Encyclopedia of mathematics education (pp. 255–260). New York: Springer.

[ref7] Blanton M. Isler-Baykal I. Stroud R. Stephens A. Knuth E. Gardiner A. M. (2019). Growth in children’s understanding of generalizing and representing mathematical structure and relationships. Educ. Stud. Math. 102, 193–219. doi: 10.1007/s10649-019-09894-7

[ref8] Blanton M. L. Kaput J. J. (2011). “Functional thinking as a route into algebra in the elementary grades,” in Early Algebraization. Advances in Mathematics Education, eds. Cai J. Knuth E. (Berlin, Heidelberg: Springer).

[ref9] Braun V. Clarke V. (2006). Using thematic analysis in psychology. Qual. Res. Psychol. 3, 77–101. doi: 10.1191/1478088706qp063oa

[ref9004] Braun V. Clarke V. (2021). Can I use TA? Should I use TA? Should I not use TA? Comparing reflexive thematic analysis and other pattern‐based qualitative analytic approaches. Couns. Psychother. Res. 21, 37–47. doi: 10.1002/capr.12360

[ref9005] Braun V. Clarke V. (2023). Toward good practice in thematic analysis: Avoiding common problems and be (com) ing a knowing researcher. Int. J. Transgender Health. 24, 1–6. doi: 10.1080/26895269.2022.2129597 PMC9879167 36713144

[ref10] Brenner M. E. Mayer R. E. Moseley B. Brar T. Durán R. Reed B. S. . (1997). Learning by understanding: the role of multiple representations in learning algebra. Am. Educ. Res. J. 34, 663–689. doi: 10.3102/00028312034004663

[ref11] Bütüner S. Ö. Baki A. The A. (2020). The use of history of mathematics in the mathematics classroom: an action study. Int. J. Educ. Math. Sci. Technol. (IJEMST) 8, 92–117.

[ref12] Carlsson M. O. Zou K. H. Yu C. R. Liu K. Sun F. W. (2014). A comparison of nonparametric and parametric methods to adjust for baseline measures. Contemp. Clin. Trials 37, 225–233. doi: 10.1016/j.cct.2014.01.002, 24462567

[ref13] Castro W. F. Cisneros J. W. Vergel R. (2025). Instructional design and its implementation: the case of the process of objectification of the rational number through measurement processes. Discov. Educ. 4:207. doi: 10.1007/s44217-025-00507-5

[ref14] Chorlay R. Clark K. M. Tzanakis C. (2022). “History of mathematics in mathematics education: recent developments in the field,” in ZDM–Mathematics Education, (Cham, Switzerland: Springer), 1407–1420.

[ref15] Clark K. M. (2019). “History and pedagogy of mathematics in mathematics education: history of the field, the potential of current examples, and directions for the future,” in Eleventh Congress of the European Society for Research in Mathematics Education (No. 2), (Cologne (Germany): Freudenthal Group; Freudenthal Institute; ERME).

[ref9006] Clark K. M. Kjeldsen T. H. Schorcht S. Tzanakis C. (2016). History of mathematics in mathematics education: Recent developments. In Radford L. Furinghetti F. Hausberger T. (Eds.), Proceedings of the 2016 ICME Satellite Meeting – HPM 2016 (pp. 135–179). IREM de Montpellier.

[ref16] Clark K. M. Kjeldsen T. H. Schorcht S. Tzanakis C. (2019). History of mathematics in mathematics education–an overview. Mathematica Didactica 42, 3–28. doi: 10.18716/ojs/md/2019.1374

[ref17] Connor M. B. (1956). A historical survey of methods of solving cubic equations. Master's Theses. 114. Available online at: https://scholarship.richmond.edu/masters-theses/114

[ref19] Corry L. (2003). Modern Algebra and the Rise of Mathematical Structures. Boston: Birkhäuser, Springer Science & Business Media.

[ref20] Deakin M. (2005). History of mathematics: solving cubic equations. Parabola 41, 1–5. Available online at: https://www.parabola.unsw.edu.au/sites/default/files/2024-03/vol41_no3_6.pdf

[ref21] Doğruer Ş. Ş. (2024). Gifted students’ views on integrating history of mathematics in mathematics lessons gifted students’ views on integrating history of mathematics in. Investig. Math. Learn. 17, 1–20. doi: 10.1080/19477503.2024.2409031

[ref22] Dorier J. L. Sierpinska A. (2001). “Research into the teaching and learning of linear algebra,” in The Teaching and Learning of Mathematics at University Level: An ICMI Study, (Dordrecht: Springer Netherlands), 255–273.

[ref23] Dubinsky E. (1991). “Constructive aspects of reflective abstraction in advanced mathematics,” in Epistemological Foundations of Mathematical Experience. Recent Research in Psychology, ed. Steffe L. P. (New York: Springer).

[ref24] Dubinsky E. McDonald M. A. (2001). “APOS: a constructivist theory of learning in undergraduate mathematics education research,” in The Teaching and Learning of Mathematics at University Level: An ICMI Study, (Dordrecht: Springer Netherlands), 275–282.

[ref25] Duval R. (2006). A cognitive analysis of problems of comprehension in a learning of mathematics. Educ. Stud. Math. 61, 103–131. doi: 10.1007/s10649-006-0400-z

[ref26] Eves H. (1958). Omar Khayyam's solution of cubic equations. Math. Teach. 51, 285–286.

[ref27] Farmaki V. Klaudatos N. Paschos T. (2004). Integrating the history of mathematics in educational praxis. An Euclidean geometry approach to the solution of motion problems. Proceedings of the 28th Conference of the International Group for the Psychology of Mathematics Education, Athens: Hellenic Mathematical Society / University of Athens. 3, 505–512.

[ref28] Farmaki V. Paschos T. (2007). Employing genetic ‘moments’ in the history of mathematics in classroom activities. Educ. Stud. Math. 66, 83–106. doi: 10.1007/s10649-006-9056-y

[ref29] Florio E. (2020). A synergy between history of mathematics and mathematics education: a possible path from geometry to symbolic algebra. Educ. Sci. 10:243. doi: 10.3390/educsci10090243

[ref30] Fried M. N. (2001). Can mathematics education and history of mathematics coexist? Sci. Educ. 10, 391–408. doi: 10.1023/A:1011205014608

[ref31] Fried M. N. (2014). “History of mathematics in mathematics education,” in International Handbook of Research in History, Philosophy and Science Teaching, ed. Matthews M. (Dordrecht: Springer). doi: 10.1007/978-94-007-7654-8_21

[ref32] Fried M. N. (2018). “History of mathematics, mathematics education, and the liberal arts,” in Invited Lectures from the 13th International Congress on Mathematical Education, (Cham: Springer International Publishing), 85–101.

[ref33] Gall M. D. Gall J. P. Borg W. R. (2007). Educational Research: An Introduction. 8. utg Edn). (AE Burvikovs, Red.) New York: Pearson.

[ref34] Godino J. D. Giacomone B. Batanero C. Font V. (2017). Onto-semiotic approach to mathematics teacher's knowledge and competences. Bolema 31, 90–113.

[ref35] Goktepe S. Ozdemir A. S. (2021). An example of using history of mathematics in classes. Eur. J. Sci. Math. Educ. 1, 125–136. doi: 10.30935/scimath/9392

[ref36] Guest G. Bunce A. Johnson L. (2006). How many interviews are enough? An experiment with data saturation and variability. Field Methods 18, 59–82. doi: 10.1177/1525822X05279903

[ref37] Guilbeau L. (1930). The history of the solution of the cubic equation. Math. News Lett. 5, 8–12. doi: 10.2307/3027812

[ref38] Harel G. (2013). Classroom-based interventions in mathematics education: relevance, significance, and applicability. ZDM 45, 483–489. doi: 10.1007/s11858-013-0503-9

[ref39] Harel G. (2018). “The learning and teaching of linear algebra through the lenses of intellectual need and epistemological justification and their constituents,” in Challenges and Strategies in Teaching Linear Algebra. ICME-13 Monographs, eds. Stewart S. Andrews-Larson C. Berman A. Zandieh M. (Cham: Springer).

[ref40] Harel G. Soto O. (2017). Structural reasoning. Int. J. Res. Undergrad. Math. Educ. 3, 225–242. doi: 10.1007/s40753-016-0041-2

[ref41] Hawthorne C. Druken B. K. (2019). Looking for and using structural reasoning. Math. Teach. 112, 294–301. doi: 10.5951/mathteacher.112.4.0294

[ref42] Heeffer A. (2007). “Learning concepts through the history of mathematics: the case of symbolic algebra,” in Philosophical Dimensions in Mathematics Education, (Boston: Springer US), 83–103.

[ref43] Hilton C. E. (2017). The importance of pretesting questionnaires: a field research example of cognitive pretesting the exercise referral quality of life scale (ER-QLS). Int. J. Soc. Res. Methodol. 20, 21–34. doi: 10.1080/13645579.2015.1091640

[ref44] Hoch M. (2003). Structure sense. In Proceedings of the 3rd Conference for European Research in Mathematics Education. Dordrecht: Springer. (Vol. 3, pp. 1–3).

[ref45] Hoch M. Dreyfus T. (2006). Structure sense versus manipulation skills: an unexpected result. In Proceedings of the 30th Conference of the International Group for the Psychology of Mathematics Education (Vol. 3, pp. 305–312).

[ref46] Høyrup J. (2019). “(Article II.8.) A hypothetical history of old Babylonian mathematics − places, passages, stages, development,” in Selected Essays on Pre- and Early Modern Mathematical Practice, (Cham: Springer).

[ref47] Jankvist U. T. (2009). A categorization of the “whys” and “hows” of using history in mathematics education. Educ. Stud. Math. 71, 235–261. doi: 10.1007/s10649-008-9174-9

[ref48] Kalantari B. Zaare-Nahandi R. (2022). On Tusi's classification of cubic equations and its connections to Cardano's formula and Khayyam's geometric solution. arXiv preprint arXiv:2201.13282. doi: 10.48550/arXiv.2201.13282

[ref49] Kapofu L. K. Kapofu W. (2020). “This maths is better than that maths” – exploring learner perceptions on the integration of history of mathematics in teaching the theorem of Pythagoras: a case study. Int. Electron. J. Math. Educ. 15:em0604. doi: 10.29333/iejme/8446

[ref50] Kaput J. J. (1998). Representations, inscriptions, descriptions and learning: a kaleidoscope of windows. J. Math. Behav. 17, 265–281. doi: 10.1016/s0364-0213(99)80062-7

[ref51] Kaput J. (2008). “What is algebra? What is algebraic reasoning?” in Algebra in the Early Grades, eds. Kaput J. J. Carraher D. W. Blanton M. L. (New York: Lawrence Erlbaum Associates), 235–272.

[ref52] Karp A. Furinghetti F. (2016). History of Mathematics Teaching and Learning. Cham: Springer.

[ref53] Katz V. J. (2021). The Mathematics of Egypt, Mesopotamia, China, India, and Islam: A Sourcebook. Cham: Springer.

[ref54] Katz V. J. Barton B. (2007). Stages in the history of algebra with implications for teaching. Educ. Stud. Math. 66, 185–201. doi: 10.1007/s10649-006-9023-7

[ref9008] Kent D. A. Muraki D. J. (2016). A geometric solution of a cubic by Omar Khayyam… in which colored diagrams are used instead of letters for the greater ease of learners. Am. Math. Mon. 123, 149–160. doi: 10.4169/amer.math.monthly.123.2.149

[ref55] Kieran C. (1989). A perspective on algebraic thinking. In Vernand G. Rogalski Artigue M. (Eds). Proceedings of the 13th International Conference for the Psychology of Mathematics Education (Vol. 2, pp. 163–171). Paris.

[ref56] Kieran C. (1992). “The learning and teaching of school algebra,” in Handbook of Research on Mathematics Teaching and Learning, ed. Grouws D. A. (New York: Macmillan), 390–419.

[ref57] Kieran C. (2006). “Research on the learning and teaching of algebra,” in Handbook of Research on the Psychology of Mathematics Education, 11–49.

[ref58] Kieran C. (2013). “The false dichotomy in mathematics education between conceptual understanding and procedural skills: an example from algebra,” in Vital Directions for Mathematics Education Research, ed. Leatham K. (New York: Springer).

[ref59] Kieran C. (2018). “Seeking, using, and expressing structure in numbers and numerical operations: a fundamental path to developing early algebraic thinking,” in Teaching and Learning Algebraic Thinking with 5- to 12-Year-Olds. ICME-13 Monographs, ed. Kieran C. (Cham: Springer).

[ref9009] Kieran C. Sfard A. (1999). Seeing through symbols: The case of equivalent expressions. Focus on learning problems in mathematics. 21, 1–17. Available online at: https://www.researchgate.net/profile/Anna-Sfard/publication/234603435_Seeing_through_Symbols_The_Case_of_Equivalent_Expressions/links/54113ef90cf2df04e75d73a0/Seeing-through-Symbols-The-Case-of-Equivalent-Expressions.pdf

[ref60] Kouropoulos G. (2025). Middle ages: the conflicts among mathematicians in Europe regarding the problem of the cubic and quartic equation. Available at SSRN 5447674. doi: 10.2139/ssrn.5447674

[ref61] Küchemann D. Hoyles C. (2009). “From empirical to structural reasoning: tracking changes over time,” in Teaching and Learning Proof across the Grades Hillsdale, eds. Stylianou D. A. Blanton M. L. Knuth E. J. (London; New York: Routledge), 171–191.

[ref62] Leron U. (1985). A direct approach to indirect proofs. Educ. Stud. Math. 16, 321–325. doi: 10.1007/bf00776741

[ref63] Lichy T. (2025). The History of Mathematics as a Source for Pre-Algebraic Thinking.

[ref64] Lim S. Y. Chapman E. (2015). Effects of using history as a tool to teach mathematics on students’ attitudes, anxiety, motivation and achievement in grade 11 classrooms. Educ. Stud. Math. 90, 189–212. doi: 10.1007/s10649-015-9620-4

[ref65] Linchevski L. Livneh D. (1999). Structure sense: the relationship between algebraic and numerical contexts. Educ. Stud. Math. 40, 173–196. doi: 10.1023/A:1003606308064

[ref66] Liu P. (2003). Do teachers need to incorporate the history of mathematics in their teaching? Math. Teach. 96, 416–421. doi: 10.5951/MT.96.6.0416

[ref67] Marghetis T. Landy D. Goldstone R. L. (2016). Mastering algebra retrains the visual system to perceive hierarchical structure in equations. Cogn. Res. Princ. Implic. 1:25. doi: 10.1186/s41235-016-0020-9, 28180176 PMC5256452

[ref68] Ministry of Education (MoE), National Council for Curriculum and Assessment (2023). Additional Mathematics Curriculum for Secondary Education (SHS 1–3). Accra, Ghana: Ministry of Education, Republic of Ghana.

[ref69] Nataraj M. S. Thomas M. (2016). “Teaching and learning middle school algebra: valuable lessons from the history of mathematics,” in And the Rest is Just Algebra, (Cham: Springer International Publishing), 131–154.

[ref71] Newton K. J. Star J. R. Lynch K. (2010). Understanding the development of flexibility in struggling algebra students. Math. Think. Learn. 12, 282–305. doi: 10.1080/10986065.2010.482150

[ref72] Novotná J. Hoch M. (2008). How structure sense for algebraic expressions or equations is related to structure sense for abstract algebra. Math. Ed. Res. J. 20, 93–104. doi: 10.1007/BF03217479

[ref73] Nunnally J. C. Bernstein I. H. (1994). Psychometric Theory. 3rd Edn New York: McGraw-Hill.

[ref74] Panagiotou E. N. (2011). Using history to teach mathematics: the case of logarithms. Sci. Educ. 20, 1–35. doi: 10.1007/s11191-010-9276-5

[ref75] Psillos S. (2006). The structure, the whole structure, and nothing but the structure? Philos. Sci. 73, 560–570. doi: 10.1086/518326

[ref76] Quade D. (1984). 10 nonparametric methods in two-way layouts. Handb. Stat. 4, 185–228. doi: 10.1016/S0169-7161(84)04012-8

[ref77] Radford L. G. (2001). “The historical origins of algebraic thinking,” in Perspectives on School Algebra. Mathematics Education Library, eds. Sutherland R. Rojano T. Bell A. Lins R., vol. 22 (Dordrecht: Springer).

[ref78] Radford L. Bartolini Bussi M. G. Bekken O. Boero P. Dorier J.-L. Katz V. . (2002). Historical Formation and Student Understanding of Mathematics, 143–170.

[ref9011] Radford L. (1997). On psychology of algebraic thinking. In Pehkonen E. (Ed.), Proceedings of PME 21 (Vol. 4, pp. 17–24). University of Helsinki.

[ref9010] Radford L. (2014). The progressive development of early embodied algebraic thinking. Math. Educ. Res. J., 26, 257–277. doi: 10.1007/s13394-013-0087-2

[ref79] Rashed R. (2013). Ibn al-Haytham's Theory of Conics, Geometrical Constructions and Practical Geometry: A History of Arabic Sciences and Mathematics, vol. 3, London (UK): Routledge.

[ref80] Richardson J. T. (2011). Eta squared and partial eta squared as measures of effect size in educational research. Educ. Res. Rev. 6, 135–147. doi: 10.1016/j.edurev.2010.12.001

[ref81] Sfard A. (1991). On the dual nature of mathematical conceptions: reflections on processes and objects as different sides of the same coin. Educ. Stud. Math. 22, 1–36. doi: 10.1007/BF00302715

[ref82] Sfard A. (1995). The development of algebra: confronting historical and psychological perspectives. J. Math. Behav. 14, 15–39. doi: 10.1016/0732-3123(95)90022-5

[ref83] Sfard A. Linchevski L. (1994). The gains and the pitfalls of reification — the case of algebra. Educ. Stud. Math. 26, 191–228. doi: 10.1007/BF01273663

[ref9012] Shadish W. R. Cook T. D. Campbell D. T. (2002). Quasi-experiments: interrupted time-series designs. Experimental and quasi-experimental designs for generalized causal inference. 1, 171–205. Available online at: https://www.academicpeds.org/wp-content/uploads/gravity_forms/66-cffe81f82d0c22a20931a4634aea2e41/2023/03/Shadish-Cook-Cambell_2002_-ITS_Designs_OCR.pdf

[ref84] Siadat M. V. Tholen A. (2020). Omar Khayyam. Math. Horiz. 28, 12–15. Available online at: https://www.jstor.org/stable/48664645

[ref85] Sibgatullin I. R. Korzhuev A. V. Khairullina E. R. Sadykova A. R. Baturina R. V. Chauzova V. (2022). A systematic review on algebraic thinking in education. Eurasia J. Math. Sci. Technol. Educ. 18, 1–15. doi: 10.29333/ejmste/11486

[ref86] Skemp R. R. (1976). Relational understanding and instrumental understanding. Math. Teach. 77, 20–26. doi: 10.5951/MTMS.12.2.0088

[ref87] Star J. (2020). “Instrumental and relational understanding in mathematics education,” in Encyclopedia of Mathematics Education, ed. Lerman S. (Cham: Springer).

[ref88] Stehlíková N. (2004). “Structural understanding in advanced mathematical thinking,” in Nada Stehlikova.

[ref89] Strobach P. E. T. E. R. (2016). How Do I Solve A Cubic Equation. Technical Report. Available online at: https://www.researchgate.net/publication/300009555

[ref90] Sun S. Sun D. Xu T. (2023). The developmental progression of early algebraic thinking of elementary school students. J. Intelligence 11:222. doi: 10.3390/jintelligence11120222, 38132840 PMC10744471

[ref91] Susac A. Bubic A. Vrbanc A. Planinic M. (2014). Development of abstract mathematical reasoning: the case of algebra. Front. Hum. Neurosci. 8, 1–10. doi: 10.3389/fnhum.2014.00679, 25228874 PMC4151197

[ref92] Swetz F. (1995). To know and to teach: mathematical pedagogy from a historical context. Educ. Stud. Math. 29, 73–88. doi: 10.1007/BF01273901

[ref93] Tall D. Vinner S. (1981). Concept image and concept definition in mathematics with particular reference to limits and continuity. Educ. Stud. Math. 12, 151–169. doi: 10.1007/BF00305619

[ref94] Torres M. D. Ayala-Altamirano C. Ramírez- Uclés R. (2025). Growing patterns invention by primary education students. Res. Math. Educ. 27, 566–586. doi: 10.1080/14794802.2024.2344211

[ref95] Torres M. Moreno A. Cañadas M. (2021). Generalization process by second grade students. Mathematics 9, 1–19. doi: 10.3390/math9101109

[ref96] Toscano F. (2020). The Secret Formula: How a Mathematical Duel Inflamed Renaissance Italy and Uncovered the Cubic Equation. New Jersey: Princeton University Press.

[ref97] Tzanakis C. Arcavi A. (2002). Integrating History of Mathematics in the Classroom: An Analytic Survey, 201–240.

[ref98] Zerpa L. (2016). The reification of mathematical notions in mathematics education: a four-stage model of concept development. Int. J. Sci. Math. Technol. Learn. 24:1. doi: 10.18848/2327-7971/CGP/v24i01/1-14

